# Urinary metabolomics profile in children with ureteropelvic junction obstruction

**DOI:** 10.1007/s11306-026-02527-0

**Published:** 2026-09-22

**Authors:** Lucas Henrique Ferreira da Silva, Marcos Figueiredo Mello, Felipe Guilherme Hamoy Kataoka, Umberto Fauze Amsei Filho, João Pedro Simon Farah, Lydia Fumiko Yamaguchi, Massuo Jorge Kato, Marcone Augusto Leal de Oliveira, Andréa Tedesco Faccio, Lucia Andrade, Francisco Tibor Dénes, Roberto Iglesias Lopes, Marina Franco Maggi Tavares

**Affiliations:** 1 Center for Multiplatform Metabolomics Studies (CEMM), Institute of Chemistry, University of Sao Paulo, Sao Paulo, Brazil; 2 Division of Urology, Hospital das Clínicas, University of São Paulo Medical School, Sao Paulo, Brazil; 3 Department of Surgery, Urology Unit, School of Medicine at the Federal, University of Paulo, Sao Paulo, Brazil; 4 Institute of Chemistry, University of Sao Paulo, Sao Paulo, Brazil; 5 Federal University of Juiz de Fora, Juiz de Fora, Brazil; 6 Division of Nephrology, Hospital das Clínicas, University of São Paulo Medical School, Sao Paulo, Brazil; 7 Present Address: Instituto de Pesquisas Tecnológicas do Estado de São Paulo IPT NUTABES , Sao Paulo, Brazil; 8 Present Address: Grupo Fleury , Sao Paulo, Brazil

**Keywords:** Biomarker, Metabolomics, Obstructive hydronephrosis, Ureteropelvic junction obstruction

## Abstract

**Introduction:**

Fetal hydronephrosis (HN) is detected in about 0.25–1% of fetuses, where ureteropelvic junction obstruction (UPJO) is the most commonly found congenital urinary tract anomaly, remaining the leading cause of kidney failure in infants and children. Since it is a spectral disease, UPJO represents a particularly challenging management. Severe UPJO must be treated surgically to avoid impairment of renal function, but children with non-obstructive hydronephrosis can be treated conservatively. There has been controversy regarding the indication for surgical intervention in asymptomatic patients.

**Objectives:**

This pilot study aimed at investigating urine samples from children with UPJO using gas- and high-performance liquid-chromatography coupled to mass spectrometry (GC- and HPLC-MS) via untargeted metabolomics to prospect discriminatory metabolites indicative of hydronephrosis spectral progression that may contribute to optimize clinical decision-making.

**Methods:**

Thirty-seven patients, whose clinical characteristics were recorded upon visits, had urinary samples collected, processed and analyzed by both GC- and HPLC-MS analytical platforms. Three distinct age-matched groups (2–12 years old) were inspected: 10 children with obstructive HN (OHN) established at initial imaging diagnosis, 15 asymptomatic children with non-obstructive HN (NOHN), and 12 children without any urinary tract problems as control group (CTR). Univariate analysis (including distribution checking and goodness-of-fit testing using ANOVA, Tukey test, and Benjamini-Hochberg procedure for FDR estimation) and multivariate analysis (PLS-DA, using cross-validation and permutation testing) were applied to the metabolomics data. Exploratory ROC curve analyses were performed using combinations of PLS-DA ranked features with VIP > 1 to assess their discriminatory performance.

**Results:**

Metabolomics profiling revealed distinct expression patterns upon selected group comparisons. The inspection of discriminatory metabolites across the different study groups allowed a clear visualization of the disease progression at the molecular level. Moreover, the discrimination of asymptomatic patients (NOHN group) from those requiring surgical intervention (OHN group) is of upmost relevance and could also be delineated here. All univariate and multivariate methods applied to both GC- and HPLC-MS data exhibited good statistical performance (adjusted *p*-values < 0.05, 0.04 < *q*-values < 0.1, and 0.662 < AUC < 0.882 for univariate analysis; *p*-value < 0.03, 3 < F < 11.4, 0.984 < R^2^<0.996, 0.714 < Q^2^<0.859, and 0.923 < AUC < 0.995 for multivariate analysis) resulting in a total of 94 discriminatory metabolites, suggesting strong potential clinical value as a diagnostic/prognostic signature. Worth mentioning cystine and methionine sulfoxide exhibiting the largest positive fold change scores, 156% and 152%, respectively (both from OHN vs. CTR group comparison). From the total of 94 discriminatory metabolites, 17 metabolites had particular statistical relevance since they were confirmed by both univariate and multivariate methods combined. Three metabolites, 1-methylhistidine-3-methylhistidine, 1-methyhistamine-3methylhistamine, and glutamyl-hydroxyproline (dipeptide), discriminate NOHN vs. CTR groups whereas nine metabolites, mandelic acid, pantothenic acid, furoylglicine, gluconic acid, 3-hydroxyphenylacetate, 4-hydroxyphenylacetate, glutamylhydroxyproline, 3-hydroxy-3-methylglutaric acid, ribitol/arabitol/xylitol (pentose alcohols) and tagalose discriminate OHN vs. CTR groups; those metabolites may serve as diagnostics purposes, being the first group comparison (NOHN vs. CTR) indicative of the initial metabolic perturbation caused by the UPJO and the latter group comparison (OHN vs. CTR) indicative of the impact UPJO had exerted on the children metabolism at long term. More importantly, five metabolites, ascorbic acid, furoylglycine, gluconic acid, and ribose/arabinose/xylose (pentoses), already discriminating OHN vs. CTR groups, appear again in the OHN vs. NOHN group comparison, in addition to threitol; those metabolites might be useful to support clinical decisions whether a patient should undergo surgery. Metabolomic pathway analyses revealed an alteration of the beta-alanine metabolism at early stages of HN, progressing to further compromising of tyrosine, phenylalanine, alanine/aspartate/glutamate, and amino sugar/nucleotide sugar metabolisms. Several other important metabolic pathways were further compromised at advanced stages of HN revealing the overall impact of UPJO at the basal metabolism of healthy children.

**Conclusion:**

Despite the limited size and age spanning of the cohort evaluated here, the statistical quality of the present study, that certified 17 metabolites by both univariate and multivariate methods among 94 discriminant metabolites, deriving from NOHN, OHN, and CTR group comparisons, may contribute to pave the way into searching valuable biomarkers for the obstructive HN setting up and progression.

**Supplementary Information:**

The online version contains supplementary material available at https://doi.org/10.1007/s11306-026-02527-0.

## Introduction

Fetal hydronephrosis is detected in about 0.25–1% of fetuses by antenatal ultrasonographic screening (Mesrobian et al., 2012), where ureteropelvic junction obstruction (UPJO) is the most commonly found congenital urinary tract anomaly, with a prevalence of 1 in 1500 live births, remaining the leading cause of kidney failure in infants and children (Chevalier & Peters, (Chevalier & Peters, 2002). Since it is a spectral disease, UPJO represents a particularly challenging management and approximately 20% of children with this anomaly will require surgical intervention(Lam et al., 2007). In this scenario, not all hydronephrosis (HN) represents a harmful state for the kidneys. Severe UPJO must be treated surgically to avoid impairment of renal function, but children with non-obstructive hydronephrosis (NOHN) can be treated conservatively (Heinlen et al., 2009). There has been controversy regarding the indication for surgical intervention in asymptomatic patients. Some authors have proposed initial non-operative management along with intensive imaging protocols. Surgical intervention is indicated primarily on decreased ipsilateral differential renal function or increased drainage interval on nuclear scans (Koff, 2000); Lee & Han 2009).

Diagnostic methods currently used as reference standards to detect relevant UPJO are unsatisfactory and there is a great demand for non-invasive and reliable tests to predict which patient requires surgical intervention at early stages. To achieve this goal, researchers have evaluated the morphological changes associated with urinary obstruction, including tubular dilation and atrophy, thickening of the basement membrane, and interstitial fibrosis (Chevalier et al., 2010). A few prospective disease-related biomolecules, such as retinol-binding protein-4 (RBP-4), neutrophil gelatinase-associated lipocalin (NGAL), kidney injury molecule-1 (KIM-1), transforming growth factor beta-1 (TGF- β1), monocyte chemoattractant protein-1 (MCP-1), endothelin-1 (ET-1), as well as epidermal growth factor (EGF) have all been screened as potential urinary biomarkers for obstructive hydronephrosis (OHN) (Madsen et al., 2013). However, due to modest results and limited availability, none of them have been incorporated into clinical practice.

Metabolomics is a methodological approach that investigates in a comparative manner the entire set of low molecular-mass metabolites expressed by an individual or organism (metabolome) in pre-selected conditions (Aderemi et al., 2021). Both formats, untargeted (hypothesis generating) and targeted (hypothesis driven) metabolomics have the potential to function as a diagnostic/prognostic tool that can support clinical decisions regarding patient treatment. Metabolomics has been applied to several areas of medicine, urology in special, addressing several aspects of renal diseases, including the differentiation of end-stage renal disease from chronic kidney disease CKD (Dahabiyeh et al., 2023), the prognosis of acute kidney injury (AKI) (Patschan et al., 2023), and the inspection of patients with normoalbuminuric diabetic kidney disease (Qian et al., 2020), to cite a few. Pediatric renal diseases under the scrutiny of metabolomics have been revised recently, including AKI, kidney transplantation, CKD, renal dysplasia, vesicoureteral reflux, and lithiasis (Riccio et al., 2022).

The urinary proteome signature of UPJO was first investigated by Mishak group in 2006, using capillary electrophoresis coupled to mass spectrometry (CE-MS) (Decramer et al., 2006). Since this pioneer work, a few transcriptomics and proteomics studies followed (Devarakonda et al., 2020, Oktar et al., 2022, Liu et al., 2022). Specifically concerning metabolomics, there are studies involving targeted and untargeted analysis using either nuclear magnetic resonance spectroscopy (NMR) or separation hyphenated-MS techniques. A panel of 15 metabolites, including creatinine, tryptophan, choline, and aspartate have been monitored by targeted metabolomics with hydrophilic-interaction liquid- chromatography coupled to mass spectrometry (HILIC-MS) in the serum of neonates and infants as an attempt to differentiating patients that require surgery from those following systematic monitoring, revealing significant metabolite perturbations (Pavlaki et al., 2020). In an untargeted metabolomics study, NMR spectra were acquired from urine of newborns with prenatally diagnosed unilateral renal pelvis dilatation and healthy controls to identify specific urinary biomarkers for UPJO (Scalabre et al., 2022). Two main metabolic pathways were found compromised in this study, amino acid and betaine metabolisms.

The present work aimed at investigating the urinary profile of children with UPJO (including asymptomatic patients) using an untargeted metabolomics approach supported by GC-MS and HPLC-MS (reversed-phase mode) data to prospect metabolites for discriminating OHN from NOHN patients and both from healthy individuals. To our knowledge, no categorical untargeted metabolomics study, using multiplatform analytical methods to enhance the metabolome coverage, has been applied to UPJO so far. Moreover, the discrimination at the molecular level of asymptomatic patients from those requiring surgical intervention is of upmost relevance and it has been investigated here as well.

## Materials and methods

### Cohort & metabolomics grouping

A prospective observational cohort study was performed at the Hospital das Clínicas da Faculdade de Medicina da Universidade de São Paulo (Brazil) and approved by the local ethics committee (protocol 62235816.8.0000.0068). This study was carried out between September 2016 and June 2019 and comprised 37 children who had unilateral NH due to UPJO and controls, categorized by age in groups from 2 to 12 years old, according to the Food and Drug administration criteria (General) All children’s caregivers were interviewed andsigned out an informed consent form attesting to the children participation in the study.

Patients were initially evaluated (according to our examination protocol) and then followed-up for at least 6 months at regular clinical visits, when renal ultrasonography (US) and radionuclide examinations were registered and urine samples collected. Age, age at diagnosis, gender, and renal side were recorded, and at each reassessment, blood pressure and general physical conditions recorded as well.

The diagnosis of UPJO was initially suggested by renal ultrasound, with measurement of the anteroposterior pelvic diameter (APD), and the degree of hydronephrosis was graded according to the Society for Fetal Urology (SFU) classification on renal US (Onen, 2007). To confirm the diagnosis, radioisotope renal scans were performed, and the standards used were static renogram (DMSA radionuclide renal scans) and diuretic renography (DTPA radionuclide renal scans). DMSA was interpreted as differential renal function (DRF), where a dilation of the affected kidney less than 40% was considered abnormal. DTPA curves were classified according to Lee (Banker et al., 2023).

The indication for surgical intervention (pyeloplasty) was based on available guidelines (Radmayr et al., 2019; Arora et al., 2015); Olsen & Rawasdeh, 2016). Symptomatic obstruction (low back pain, urinary tract infection, urinary stones, or hematuria), impaired DRF (less than 40%), more than 10% decrease in renal function on subsequent investigations, drainage function deficiency after diuretic administration, increased DPA or worsening of hydronephrosis to SFU grades III and IV on US constitute the current proposed indications for surgical intervention (Olsen & Rawasdeh, 2016).

Exclusion criteria included associated anomalies, such as vesicoureteral reflux, obstruction of the ureterovesical junction and obstruction of the posterior urethral valves, bilateral NH, previous operation of the urinary system and other deformations of the external genital organs, deformations in the lower part of the ureter, bladder and urethra, urinary stones, urinary tract infection and neurogenic bladder dysfunction.

For the metabolomics study, patients were divided into three groups based on clinical and imaging findings, including APD, DRF, and drainage curve on radioisotope renal scintigraphy. The groups were classified as: (a) OHN group: children with unilateral OHN due to UPJO who underwent dismembered pyeloplasty using the Anderson-Hynes method; (b) NOHN group: children considered for nonoperative treatment of unilateral UPJO (clinically asymptomatic and stable according to imaging studies during follow-up); and (c) Control Group (CTR): healthy children, paired by age and sex with no underlying pathologies. The OHN group was composed of 10 children (4 boys, 6 girls; median age 4.9; 2–10 years old). The NOHN group included 15 children (9 boys, 6 girls; median age 6.9; 2–10 years old). The CTR consisted of 12 children without any urinary tract problems (8 boys, 4 girls; median age 6.3; 3–10 years old).

### Sample collection and analysis

Urine samples were collected according to the patient age and ability to urinate spontaneously, with preference for midstream samples (when possible). Children who were not toilet trained had their urine collected using a collection bag, and some patients, who had difficulty collecting in the pre-operative period, had their urine collected after anesthetic induction through bladder catheterization. Urinary culture was mandatory to exclude active urinary infection. In the OHN group, urine samples were taken before surgical repair of the UPJO. In the NOHN group, voided urine samples were collected at the time of diagnosis. For the control group, urine samples were also collected only once. Urine was collected aseptically, mixed, and centrifuged [3,000 rpm, 10 min]; the uppernatant was stored at − 80 °C.

For GC-MS analysis, urine samples were thawed and vortexed. A 100 µL aliquot was taken and transferred to an Eppendorf tube. Samples were treated with urease solution (10 mg/mL final concentration) and the tube was placed in a thermostatic bath at 37 °C for 1 h. After the treatment with urease, cold isopropanol (10 °C) in a 1:6 urine: isopropanol proportion was added for protein precipitation. Samples were again vortexed and placed in a freezer (−20 °C) for 30 min. Samples were then centrifuged at 12,000 rpm for 10 min at 4 °C. Exactly 200 µL supernatant were removed and transferred to 300 µL inserts and evaporated in an Eppendorf concentrator plus (Hamburg, Germany) in two cycles of 20 min each at 35 °C.

Further sample treatment steps include derivatization reactions. For the methoxymation reaction, 20 µL methoxyamine hydrochloride in pyridine (15 mg/mL) were added to each insert containing the urease-treated urine (dried sample). The inserts were placed in 2 mL vials, vortexed and submitted to ultrasound for 10 s. The vials were covered with aluminum foil and kept for 16 h at room temperature and protected from light. For the silylation reaction, after this 16 h period, 20 µL BSTFA (N, O-bis-(trimethylsilyl)trifluoroacetamide) with 1% TMCS (chlorotrimethylsilane) were added to each sample; samples were again homogenized and placed in a bath at 70 °C for 1 h. Samples were then diluted in 100 µL heptane and analyzed by GC-MS.

GC-MS analyses were conducted in a gas chromatograph coupled to a quadrupole mass spectrometer (Shimadzu GC-2010 Plus, Barueri, Brazil). Exactly 1 µL of the derivatized sample was injected into a HP5-MS column with dimensions: 30 m length, 0.25 mm internal diameter and 0.25 μm chemically bonded film (5%-phenyl-methylpolysiloxane stationary phase) from Agilent Technologies (Santa Clara, U.S.A.). The injector temperature was set at 250 °C and the split ratio was 1:10. Helium was used as carrier gas at a constant flow rate of 1 mL/min. The oven was kept initially at 60 °C for 1 min and raised to 300 °C at a rate of 10 °C/min gradient and held for 10 min before cooling-down. The mass spectrometer was operated in full-scan mode with a scan range of 50–600 *m/z* at a rate of 2 spectra/s. Temperatures at the transfer line, filament source and quadrupole were 290, 230 and 150 °C, respectively. Electron ionization was carried out at 70 eV.

For the HPLC-MS analysis, 100 µL sample aliquots were diluted in 300 µL methanol containing *p*-fluorophenylalanine (stock at 66.7 µmol/L; 50.0 µmol/L final concentration in urine). Samples were then stored at −20 °C for 30 min. After this period, samples were centrifuged at 1200 rpm for 10 min at 4 °C and the uppernatants separated and injected (0.50 µL) into the equipment (Agilent 1260 Infinity II chromatograph coupled online via electrospray ionization interface (ESI, positive ionization) to a Agilent 6530 mass spectrometer with a quadrupole time-of-flight mass analyzer, Q-TOF). A Phenomenex Kinetex^®^ PFP column (150 × 2.1 mm, 2.6 μm film) column maintained at 40 °C was used. Mobile phase was comprised of solvent A (0.10% v/v formic acid in water) and solvent B (0.1% v/v formic acid in methanol), eluted in gradient: 0–1 min (0% B), 1–2.5 min (0–25% B), 2.5–3 min (25–90% B), 3–5 min (90–100% B), 5–8 min (100% B), 8–8.1 min (100–0% B), 8.1–17.5 min (0% B). Voltages were optimized as follows: capillary voltage, 3500 V; fragmentor voltage, 125 V; skimmer voltage, 65 V; nozzle voltage, 1000 V; and octopole voltage, 750 V. The nebulizer pressure was set at 30 psi and the desolvation gas flows was maintained at 250 °C and 8 L/min. The sheath gas was operated at 11 L/min and 350 °C. For mass accuracy, purine (121.0509 *m/z*; 5.0 µmol/L in 95% acetonitrile) and HP-0921 ((hexakis(1 H,1 H,3 H-tetrafluoropropoxy)phosphazene); 922.0098 *m/z*; 2.5 µmol/L in 95% acetonitrile) were infused as a lock masses via an auxiliary sprayer at 3 µL/min. Data were acquired in centroid mode across a scan range of 75–1200 *m/z* at a rate of 1 spectrum/s.

A quality control (QC) sample was prepared for each technique. For this purpose, 15 µL of all urine samples under consideration in this study were mixed together, separated into two portions and processed exactly by the same procedures individual urine samples did. Three samples containing only water were subjected to urease treatment and subsequently to derivatization steps and analyzed as a prepared blank solution in GC-MS. QC samples were injected sequentially, one QC injection after every five regular samples.

### Statistical analysis

Statistical analysis was performed using IBM SPSS Statistics for Windows, version 17.0 (SPSS, Chicago, IL, U.S.A.), SIMCA *P* + 12.0.1 and the free access software packages, XCMS in the R platform (R Project for Statistical Computing, v.4.4.2) and MetaboAnalyst 6.0.

## Results

Demographic characteristics and clinical data of the patient and control groups under investigation here are presented in Table 1. All patients had a normal estimated glomerular filtration rate (eGFR) > 90 ml/min/1:73m^2^ calculated using the Schwartz formula (Schwartz and Work 2009). In the OHN group, 4 patients were symptomatic: 2 had low back pain, 1 with febrile UTI, and 1 with non-febrile UTI; in the NOHN group, 2 patients were symptomatic, all had non-febrile UTI that resolved on follow-ups. There was a statistically significant difference between the groups in relation to symptoms, degree of hydronephrosis on the SFU classification scale in the US, DRF on DMSA, and pattern of renal scan curves on DTPA.

**Table 1:** Demographic and clinical characteristics parameters of the patient and control groups

Parameter	OHN group	NOHN group	CTR group
Age (years) ^b^	4.9 (2–12)^†^	6.9 (2–12)^†^	6.3 (3–10)^†^
Sex (male/female) ^b^	4/6	9/6	8/4
Laterality (right/left) ^b^	5/5	4/11	NE
Congenital HN ^b^	6 (60%)	8 (53%)	NE
Symptomatic ^a^	4 (40%)	2 (13%)	NE
APD (mm) ^a^	31.2 (16–65) ^†^	19.7 (7–40) ^†^	NE
SFU grade IV ^a^	6 (60%)	0	NE
Split renal function ^a^	37.7 (11–57) ^†^	48.4 (40–55) ^†^	NE
Lee curve IV ^a^	10 (100%)	0	NE

*APD* anterior-posterior diameter of affected renal pelvis, *HN *hydronephrosis; NE, not evaluated

^†^ values listed as average and in parentheses the minimum and maximum values

^a^*p* < 0.05 between OHN and NOHN groups

^b^*p* > 0.05 between OHN, NOHN and CTR groups

In this cohort of the NOHN group, patients with the largest anteroposterior pelvic diameters (APD, range 21–40 mm) exhibited differential renal function (DRF) values tightly clustered between 41 and 49%, indicating essentially symmetric, normal split renal function with no cases of supranormal function among the largest-APD patients; only one patient in the entire cohort approached the supranormal threshold (DRF 55%), but with a comparatively modest APD of 20 mm. Society for Fetal Urology (SFU) grading was skewed toward milder hydronephrosis, with grades I and II accounting for 66.7% of cases and grade III for 33.3%, with no grade IV cases observed. Only one patient, presenting with an APD of 21 mm, DRF of 41%, and SFU grade III, approached a borderline profile that might prompt discussion of surgical threshold, though this patient was ultimately managed conservatively, as were all others, who likewise demonstrated clinical and radiological stability throughout the follow-up period.

All ten children in the OHN group underwent surgical intervention (Anderson-Hynes dismembered pyeloplasty) based on the criteria described previously. The indications for pyeloplasty were: 4 patients due to symptomatic obstruction, 3 patients due to impaired DRF (less than 40%), and 3 patients due to a decrease in more than 10% renal function on subsequent investigations or worsening of hydronephrosis to SFU grades III and IV on US, due to poor drainage function following diuretic administration on DTPA radionuclide renal examinations.

### Multivariate analysis

Multivariate analysis was performed using unsupervised (principal component analysis, PCA) and supervised methods (partial least-squares discriminant analysis, PLS-DA) for both GC- and LC-MS acquired data. For both instrumental techniques, PCA was first applied to the overall data including QC samples to reveal separation patterns, trends and outliers. QC samples were clustered at the center of the PCA ellipsoid plot (not shown) indicating that there was instrumental stability during data acquisition ensuring reliability for further data treatment. PLS-DA was then applied to group comparisons (NOHN vs. CTR, OHN vs. NOHN, and OHN vs. CTR) to reveal the discriminant metabolites based upon the most important variables for separation (VIP, variable importance in projection).

**Fig. 1:**
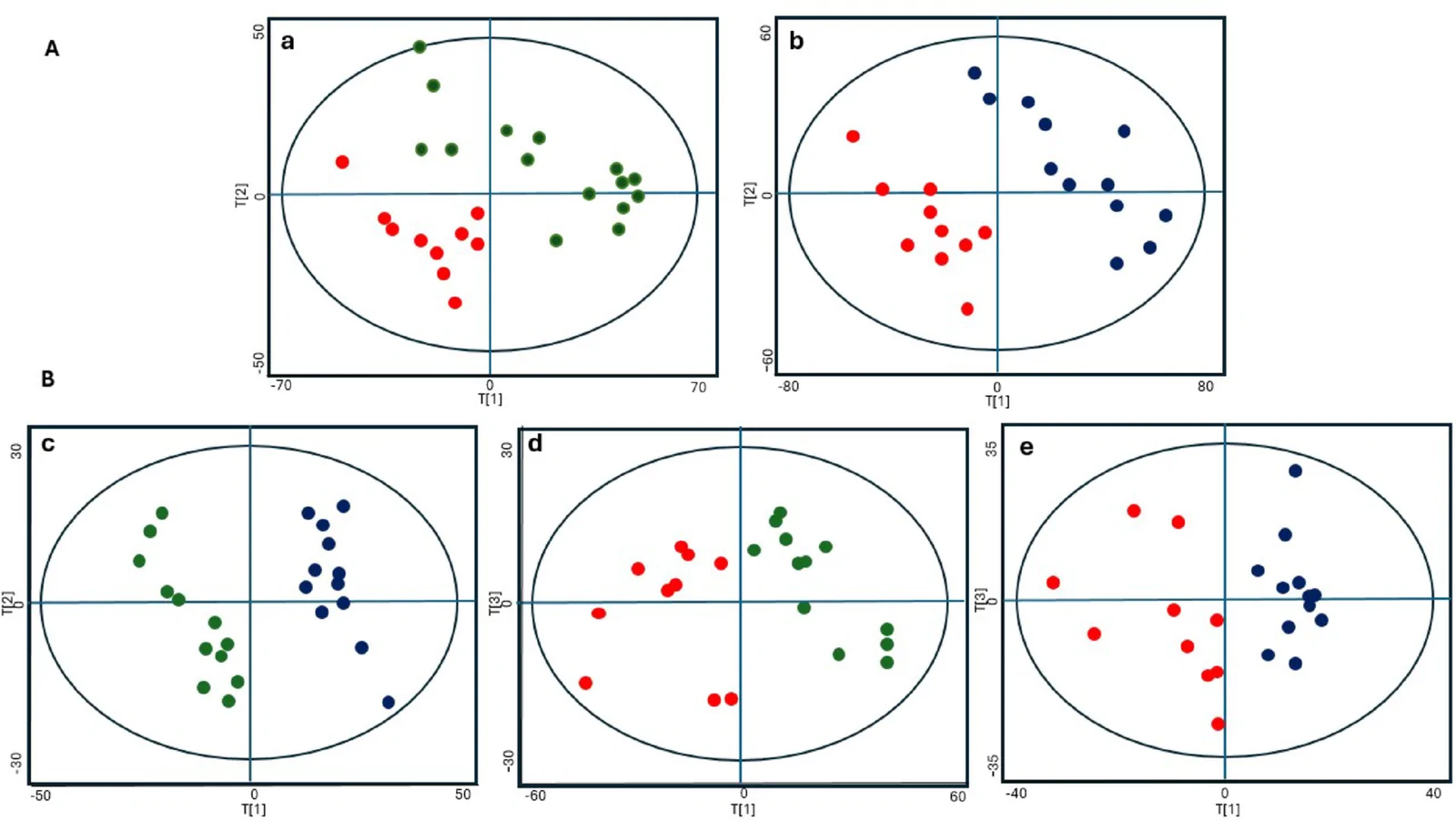
PLS-DA score plots for GC-MS and HPLC-MS acquired data. **A** GC-MS: (a) OHN vs. NOHN, R^2^ = 0.996, Q^2^ = 0.759, p-value = 0.03, F = 3 and (b) OHN vs. CTR, R^2^ = 0.91, Q^2^ = 0.743, p-value = 0.0001, F = 11.4; **B** HPLC-MS: (c) NOHN vs. CTR, R^2^ = 0.988, Q^2^ = 0.849, p-value = 0.002, F = 4.9, (d) OHN vs. NOHN, R^2^ = 0.985, Q^2^ = 0.714, p-value = 0.01, F = 4.43, and (e) OHN vs. CTR, R^2^ = 0.984, Q^2^= 0.772, p-value = 0.006, F = 4.5

Figure 1 depicts PLS-DA score plots for both GC- and HPLC-MS acquired data and corresponding model statistics for the comparisons NOHN vs. CTR, OHN vs. NOHN, and OHN vs. CTR. The risk of overfitting is always a concern for small and even large cohorts in any metabolomics study using PLS-DA models (limitation of omics studies, this work included). This occurs because sample sizes (variables) are usually small compared to the number of molecular features generated by the analytical platforms. A typical metabolomics study includes tens to hundreds of samples and generates thousands to tens of thousands of molecular features. To minimize the risk of overfitting, during the building of the PLS-DA model and generation of VIPs, both permutation and cross-validation tests were conducted. SIMCA-P uses *7-fold* cross-validation iteratively testing for optimal number of latent variables (hyperparameters) without memory of the testing sets, minimizing thus overfitting. Other relevant parameters to evaluate the PLS-DA model quality are Q^2^, which demonstrates the model’s ability to predict which group the sample belongs to, and R^2^, which represents the proportion of variance explained by the model. All models, including the ANOVA of cross-validation residuals (CV-ANOVA), presented acceptable values, except for the comparison NOHN vs. CTR via GC-MS data (CV-ANOVA *p*-value = 0.2 not shown), highlighting the importance of using more than one technique for metabolomics analysis. A permutation test was carried out using 200 permutations and no permutation Q^2^ was greater than that obtained for the original model, reinforcing the validity of the original model (Szymanska et al., 2011).

**Fig. 2:**
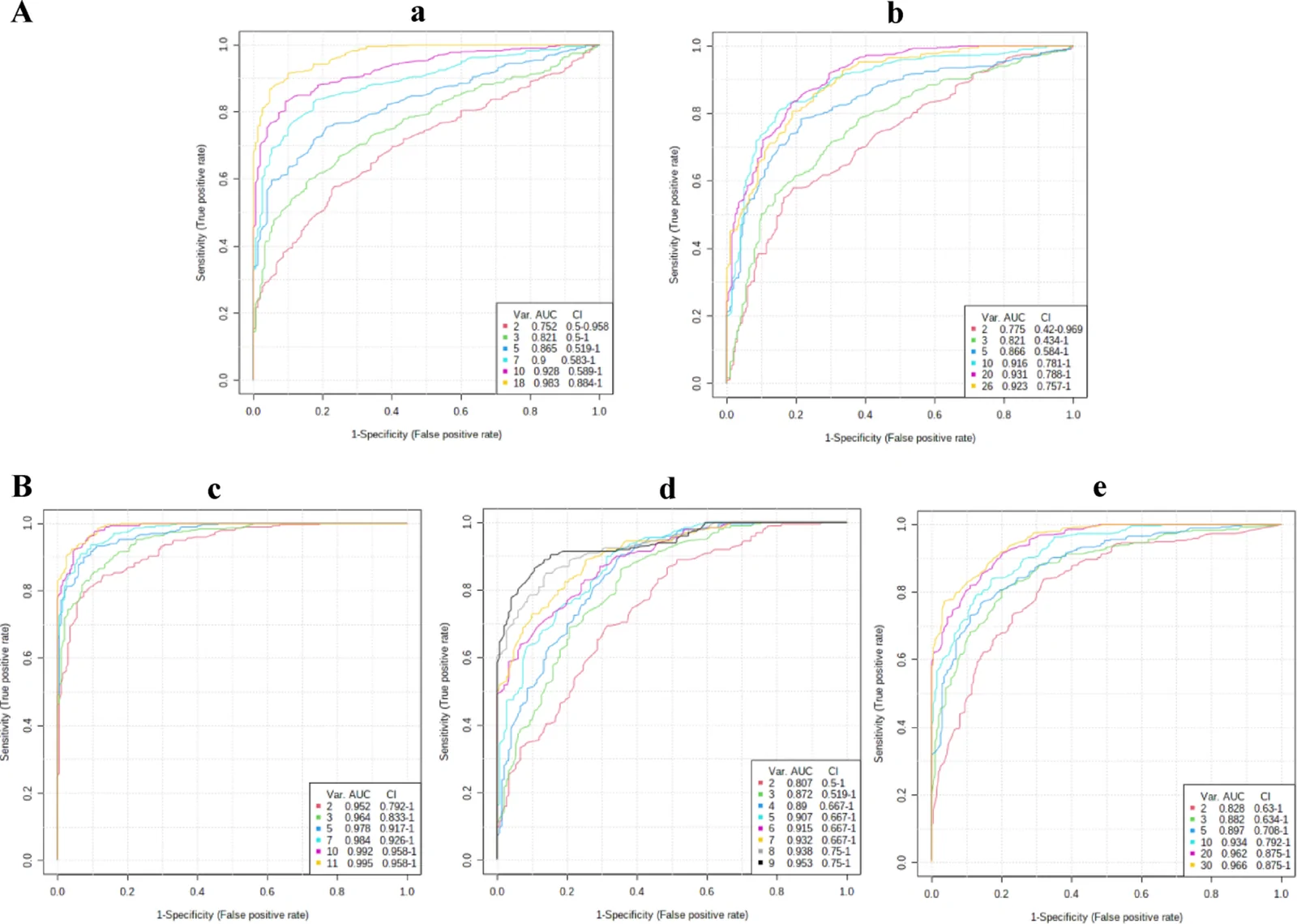
Exploratory ROC curve analyses based on PLS-DA-ranked features obtained by group comparison using GC-MS and HPLC-MS acquired data. **A** GC-MS: (a) OHN vs. NOHN, 18 metabolites, AUC = 0.983 and (b) OHN vs. CTR, 26 metabolites, AUC = 0.923; **B** HPLC-MS: (c) NOHN vs. CTR, 11 metabolites, AUC = 0.995, (d) OHN vs. NOHN, 9 metabolites, AUC = 0.953, and (e) OHN vs. CTR, 30 metabolites, AUC = 0.966

Using the MetaboAnalyst platform, ROC curves were plotted for the molecular features successfully identified as significant (VIP > 1) by the PLS-DA model and also biologically plausible to evaluate the extent of their discriminatory performance. VIP values were first ranked (the hierarchical position of resources, ranking feature), and the corresponding ROC curves plotted for increased number of VIPs. Figure 2 depicts the ROC curves for specific PLS-DA models obtained by treating GC-MS and LC-MS data, including areas under the curve (AUC) and confidence intervals (CI). As expected the discriminatory performance increased with the number of VIPs included in the computation. The satisfactory results shown in Fig. 2 for all group comparisons demonstrate the excellent ability of these sets of VIPs to distinguish between test and control groups.

### Univariate analysis

The Shapiro-Wilk normality test was applied, followed by the Bartlett test, for normal distribution data, to distinguish between equal and different variances. The Tukey’s post-hoc test was applied to identify which group comparisons were significantly different while controlling the family-wise type I error rate by adjusted *p*-values. When the assumption of normality was not met, the non-parametric Kruskal–Wallis test was performed, followed by Dunn’s post-hoc test to identify differences among the groups. In addition, the Benjamini-Hochberg method as an estimate of the false discovery rate (FDR) was performed here to address the possibility of false-positive findings resulting from multiple testings in metabolomics analysis. ROC curves were then plotted to determine the ideal cutoff point (using the Youden index) for each significant molecular feature found previously by multivariate analysis (not shown). All statistical parameters (adjusted *p*-values, *q*-values, and AUC) were listed in the Supplementary Material tables. In the multivariate approach, it was not possible to find separation patterns between NOHN vs. CTR groups for the analyses performed by GC-MS. However, when conducting an investigation using the univariate approach, significant metabolites were identified. This highlights the complementarity of multivariate and univariate statistical tools.

### Identification of metabolites

Only molecular features that presented relative standard deviation of peak intensities smaller than 30% (for QC samples) were considered for identification. For GC-MS, the molecular features that showed high spectral similarity with the NIST MS Seach 2.0 library and/or Fiehn library (Score > 80) were identified (level 1 according to Metabolomics Standards Initiative, MSI). Fiehn library also takes into account the retention time match of the derivative with the library standard. For HPLC-MS, molecular features were putatively annotated (level 2 according to MSI) searching the exact molecular mass into available databases (HMDB, Metlin, KEGG, among others). A complete set of the identified metabolites are compiled in the supplementary material (Table S1 for the comparison NOHN vs. CTR, Table S2 for the comparison OHN vs. NOHN, and Table S3 for the comparison OHN vs. CTR, displaying both GC-MS and HPLC-MS techniques results as well as univariate and multivariate parameters. All univariate and multivariate methods applied to both GC- and HPLC-MS data exhibited good statistical performance (Q^2^ > 0.71, accuracy > 92%, and AUC > 0.92) resulting in a total of 94 discriminatory metabolites, suggesting strong potential clinical value as a diagnostic/prognostic signature. A more concise display containing only the most relevant metabolites in terms of statistical relevance (when they appear by both univariate and multivariate analyses), fold change (percentage increase or decrease of a given metabolite in one studied test group related to the control or reference group), and associated metabolic routes are depicted in Table 2 for the same group comparisons. Worth mentioning cystine and methionine sulfoxide exhibiting the largest positive fold change score, 156% and 152%, respectively (both from OHN vs. CTR group comparison). Moreover, from the total of 94 discriminatory metabolites, 17 metabolites had particular statistical relevance since they were confirmed by both univariate and multivariate methods jointly. Three metabolites, 1-methylhistidine-3-methylhistidine, 1-methyhistamine-3mthylhistamine, and glutamyl-hydroxyproline (dipeptide), discriminate NOHN vs. CTR groups whereas nine metabolites, mandelic acid, pantothenic acid, furoylglicine, gluconic acid, 3-hydroxyphenylacetate, 4-hydroxyphenylacetate, glutamylhydroxyproline, 3-hydroxy-3-methylglutaric acid, ribitol/arabitol/xylitol (pentose alcohols), and tagalose discriminate OHN vs. CTR groups; those metabolites may serve as diagnostics purposes, being the former group comparison indicative of the initial metabolic perturbation UPJO imparted and the latter group comparison indicative of the impact UPJO had exerted on the children metabolism at long term. More importantly, five metabolites, ascorbic acid, furoylglycine, gluconic acid, and ribose/arabinose/xylose (pentoses), already discriminating OHN vs. CTR groups, appear again in the OHN vs. NOHN group comparison, in addition to threitol; those metabolites might be useful to support clinical decisions whether a patient should undergo surgery.

**Table 2:** Selected discriminant metabolites indicated by univariate and multivariate analysis using both GC-MS and HPLC-MS acquired data

Metabolite	Fold change, %		Group comparison		Metabolic pathway
*N*-acetyl-*L*-aspartic acid	↑	73***	OHN vs. NOHN		Alanine aspartate and glutamate metabolism
Glutamyl-hydroxyproline	↑	73	OHN vs. CTR^#^		Aminoacid degradation pathway
Alpha-glucosamine 1-phosphate	↑	48	OHN vs. NOHN		Amino sugar and nucleotide sugar metabolism
*N*-acetyl-D-mannosamine	↓	38	OHN vs. NOHN		Amino sugar and nucleotide sugar metabolism
Mandelic acid	↑ ↑	124 137	OHN vs. NOHN OHN vs. CTR^#^		Aminobenzoate degradation
Putrescine	↑	99	NOHN vs. CTR		Arginine and proline metabolism/amino acid metabolism/glutathione metabolism
	↑	283	OHN vs. CTR		
Ascorbic acid	↑	110	OHN vs. NOHN^#^		Ascorbate and aldarate metabolism
	↑	55	OHN vs. CTR		
Beta-alanine	↓	36	OHN vs. NOHN		Beta-alanine metabolism
Pantothenic acid	↓	59	OHN vs. CTR^#^		Beta-alanine metabolism/pantothenate and CoA biosynthesis
	↓	49	NOHN vs. CTR		
Muconic acid/ 2,3-Methylenesuccinic acid*	↓	71	NOHN vs. CTR		Benzoate degradation C5-branched dibasic acid metabolism
Alpha ketoglutaric acid	↓	49	OHN vs. CTR		Citrate cycle (TCA cycle)
	↓	47	OHN vs. NOHN		
Citric acid	↓	40	OHN vs. CTR		Citrate cycle (TCA cycle)
Cystine	↑	156	OHN vs. CTR		Cysteine and methionine metabolism
Methionine sulfoxide	↑	152	OHN vs. CTR		Cysteine and methionine metabolism
3-Hydroxyhippuric acid/4-hydroxyhippuric acid/ salicyluric acid *	↑	78	NOHN vs. CTR		Fatty acid metabolism pathways of salicylate elimination
Furoylglycine	↓	53***	OHN vs. CTR^#^		Fatty acid beta-oxidation
	↓	64	OHN vs. NOHN^#^		
Cystathionine	↑	182	OHN vs. CTR		Glycine, serine and threonine metabolism
1-Methylhistidine/3-methylhistidine*	↑	101	NOHN vs. CTR^#^		Histidine metabolism
1-Methylhistamine-3-methylhistamine*	↑	97	NOHN vs. CTR^#^		Histidine metabolism
3-Hydroxy-3-methylglutaric acid (dicrotalic acid)	↓	43	OHN vs. CTR^#^		lipid metabolism/fatty acid metabolism
5-Hydroxylysine	↑	137	OHN vs. CTR		Lysine degradation
Gluconic acid	↓	39	OHN vs. NOHN^#^		Pentose phosphate pathway/carbon metabolism
	↓	54	OHN vs. CTR^#^		
3-(3-Hydroxyphenyl)propanoic acid	↑	147	OHN vs. NOHN		Phenylalanine metabolism
3-(2-Hydroxyphenyl)propanoic acid	↑	258	OHN vs. CTR		Phenylalanine metabolism/tyrosine metabolism
4-Hydroxyphenylacetic acid	↑	128***	OHN vs. NOHN		Phenylalanine metabolismo/tyrosine metabolism
3-Phenyllactic acid/ desaminotyrosine/ 4-methoxyphenylacetic acid*	↑ ↑	147 258	OHN vs. NOHN OHN vs. CTR		Phenylalanine metabolism/tyrosine metabolism
2-Methylcitric acid	↑	31	NOHN vs. CTR		Propanoate metabolism
	↑	75	OHN vs. CTR		
5-Hydroxyindoleacetaldehyde/indoleacetic acid*	↑	139	OHN vs. CTR		Tryptophan metabolism
3-Hydroxyphenylacetate	↑	132	OHN vs. CTR^#^		Tyrosine metabolism
4-Hydroxyphenylacetate	↑	108***	OHN vs. CTR^#^		Tyrosine metabolism
3-Methoxytyramine	↑	109	OHN vs. CTR		Tyrosine metabolism
4-Hydroxybenzoic acid	↑	96	OHN vs. NOHN		Ubiquinone and other terpenoid-quinone biosynthesis
4-Hydroxy-3-methylbenzoic acid	↑	137	OHN vs. CTR		NA
2-Methylbutyrylglycine	↑	141	OHN vs. CTR		NA
3-Cresotinic acid	↑	132	OHN vs. CTR		NA
Dihydroxy-1*H*-indole glucuronide	↑	109	NOHN vs. CTR		NA
Glutamyl-hydroxyproline dipeptide	↑	47	NOHN vs. CTR^#^		NA
Ribitol/arabitol/xylitol (pentose alcohol)**	↑	81	OHN vs. CTR^#^		NA
Ribose/arabinose/xylose (pentose)**	↑	114	OHN vs. NOHN		NA
Tagatose (pentose)	↓	48	OHN vs. CTR^#^		NA
Threitol	↑	57	OHN vs. CTR		NA
	↓	30	OHN vs. NOHN^#^		
Threonine and tyrosine (dipeptide)	↓	76	NOHN vs. CTR		NA

*NA* not assigned

# found by both univariate and multivariate analyses

* possible attributions for that particular adduct

** analytical method does not distinguish between isomers

*** metabolite was found by both techniques; average fold change is reported

## Discussion

In this work, several potential biomarkers that could provide information about diagnosis and prediction of renal outcome were identified. However, these potential biomarkers must have some biological plausibility. The key to understanding the pathophysiological situation is the analysis of the metabolic pathways.

Again, statistical comparisons between OHN vs. CTR groups and NOHN vs. CTR groups will provide discriminant metabolites that may serve for diagnostics purposes. This is not so important once UPJO can be assertively diagnosed by other means. Nevertheless, the metabolites from OHN vs. CTR group comparison will be indicative of the impact UPJO exerted on the children metabolism in a long term. The statistical comparison between OHN vs. NOHN groups is on the other hand quite relevant because it will provide discriminant metabolites that might be useful to support the decision whether a patient should undergo surgery. Moreover, if a single metabolite or a set of metabolites appears throughout both NOHN vs. CTR and OHN vs. NOHN group comparisons these metabolites become relevant because they might be at the same time indicative of the disease implantation as well as the disease progression. With these assumptions in mind, discriminatory metabolites between all group comparisons were examined.

**Fig. 3:**
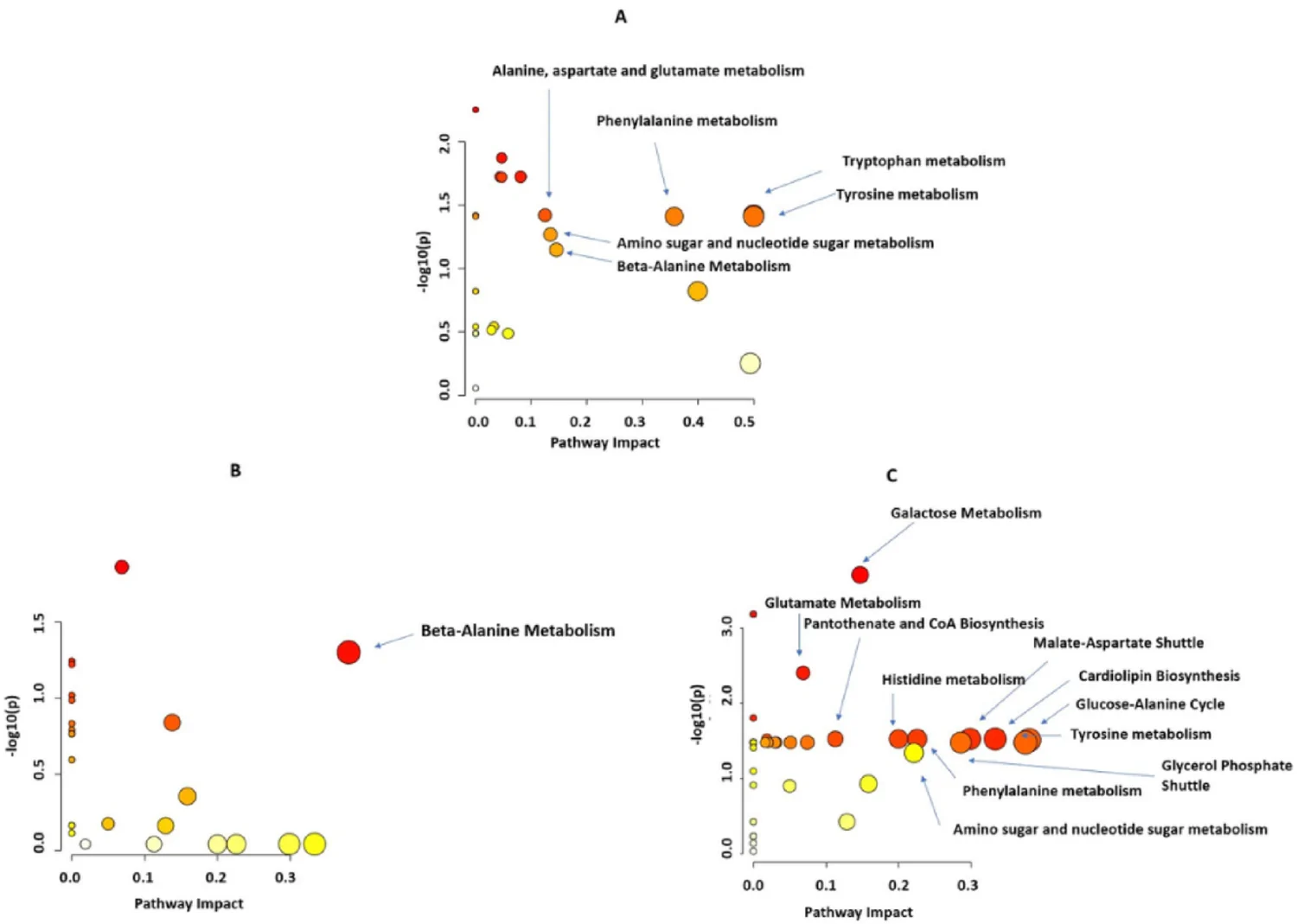
Pathway impact maps for the comparisons between OHN vs. NOHN groups **A**, NOHN vs. CTR groups **B**, and OHN vs. CTR groups **C** in UPJO

The Metabolomic Pathway Analysis (MetPA) within the MetaboAnalyst platform was used to perform topographic analysis of the metabolites identified in each group comparison (Fig. 3). Fisher’s test enrichment method was chosen, the statistical significance test in the analysis of contingency tables in the topological analysis was out-degree centrality and the library of homo sapiens metabolic pathways from KEGG database was selected. It is worth mentioning that all metabolites derived from both GC- and HPLC-MS analysis as well as both univariate and multivariate statistical approaches were considered altogether for the impact calculation that generated Fig. 3.

From inspection of Fig. 3, it is possible to visualize the most important metabolic routes associated with all group comparisons. In the NOHN vs. CTR comparison, only the metabolic pathway of beta-alanine shows a significant statistical probability. That might be explained by the fact that some obstructions are asymptomatic, not leading to renal function loss or significant health impairment. Nevertheless, although mildly, the metabolism has already been altered at this stage, which might be viewed as the disease implantation effect. The comparison of OHN vs. NOHN groups reveals several important affected metabolic routes: 1- tyrosine, phenylalanine, 2- alanine, aspartate, glutamate, and 3- amino sugar and nucleotide sugar metabolisms, as it denotes the disease progression. The comparison OHN vs. CTR groups shows the overall impact of UPJO in the metabolism, with the disease at its most severe stage, presenting the largest number of altered metabolic routes.

Although the beta-alanine metabolic pathway was the only one significantly altered in the MetPA of NOHN vs. CTR group comparison, it has also appeared in the MetPA of OHN vs. NOHN group comparison (Fig. 3B and A). This result is interesting, as it suggests that increased oxidative stress may be related to disease progression. Studies indicate that carnosine, derived from beta-alanine, plays a crucial role in protecting against oxidative stress, a factor relevant to various pathologies, including chronic kidney diseases (Calabrese et al., 2007). As observed in the metabolite compilation of Table 2, a significant fold-change decrease in beta-alanine was found (36%) for the OHN vs. NOHN comparison, indicating that this metabolite has been accumulated in the urine of the NOHN group. Pantothenic acid has also been found to be decreased in the comparison NOHN vs. CTR (−49%) and OHN vs. CTR (−59%). This metabolite is an essential precursor for the synthesis of coenzyme A (CoA) and may protect cell membranes from oxidative stress. Therefore, considering that beta-alanine pathway is altered in both MetPA of NOHN vs. CTR and OHN vs. NOHN group comparisons, it can be inferred that these alterations are associated with increased oxidative stress, which seems to intensify with disease progression. Moreover, beta-alanine derivatives exert a direct molecular protective action on podocytes, an essential part of the glomerular filtration membrane (Scuto et al., (2020).

The metabolomic profiling conducted in this study revealed further distinct expression patterns between OHN and NOHN groups (Table 2). Analysis of the urine samples suggested specific discriminators for OHN; these include 4-hydroxyphenylacetic acid, furoglycin, gluconic acid, *N*-acetyl-L-aspartic acid, and threitol. All these urinary metabolomics-based analytes had an AUC above 0.80 (Table S2), suggesting strong potential clinical value as a disease progression signature. Other metabolites also present satisfactory results should be included in the discussion to allow a more thorough evaluation of the disease progression. For instance, 4-hydroxybenzoic acid was considered statistically relevant from both univariate (AUC 0.72) and multivariate (AUC 0.923) analyses, which reinforces the importance of this metabolite in separating OHN from NOHN groups.

One of the products of the phenylalanine metabolic pathway is tyrosine. Tyrosine and phenylalanine pathways are therefore intrinsically linked to each other. In GC-MS data analysis, 4-hydroxyphenylacetic acid was identified as the major metabolite of the tyrosine pathway and 3-(3-hydroxyphenyl) propanoic acid, 4-methoxyphenylacetic acid, and 4-hydroxyphenylacetic acid as key metabolites of the phenylalanine pathway. As a matter of fact, 4-hydroxyphenylacetic acid is known to be essential for the proper functioning of the tyrosine and phenylalanine metabolic pathways. This metabolite showed the greatest expression variation in different group comparisons (increased fold change of 112% for OHN vs. NOHN and 84% for OHN vs. CTR). Its reliable identification in the database (net score of 96, Tables S2 and S3) confirms that 4-hydroxyphenylacetic acid is, in fact, a discriminatory metabolite between both NOHN vs. CTR and OHN vs. NOHN groups.

Disturbances in the metabolism of the amino acids tyrosine and phenylalanine may be related to kidney diseases (Kopple, 2007). Furthermore, in cases of chronic renal failure, there is a change in the conversion of phenylalanine into tyrosine and an increase in oxidative stress with harmful and toxic metabolic effects (Chakrapani & Holme 2006). The metabolite 4-hydroxyphenylacetic acid is increased in patients with decline in renal function (Stec et al., 2015), a glomerular disease characterized by an excessive amount of protein in the urine (proteinuria) and damage to podocytes, cells that line the urinary surface of the glomerular capillary tuft, which constitute the barrier of glomerular filtration, ensuring its selective permeability. The increased production of this antioxidant metabolite suggests a defense mechanism against increased oxidative stress. Although this metabolite has been described as a marker of glomerular pathology, our results suggest that it may also play a discriminatory role in tubular pathologies such as obstructive uropathy. It has been demonstrated that elevation of 4-hydroxyphenylacetic acid during sepsis could inhibit necroptosis of renal proximal tubule epithelial cells and exert a nephroprotective effect (Sheng et al., 2024).

In the alanine, aspartate and glutamate pathway, *N*-acetyl-*L*-aspartic acid was the most statistically significant metabolite with increased expression in the OHN group. This metabolite is associated with oxidative stress and inflammatory conditions in rats with chronic kidney disease and renal failure (Chen et al., 2017). It is also a possible serum biomarker of diabetic nephropathy (Hirayama et al., 2012).

In the amino sugar and nucleotide sugar pathway, the metabolites that stand out are alpha-glucosamine 1-phosphate (increased in OHN group vs. NOHN) and *N*-acetyl-D-mannosamine (decreased in OHN group vs. NOHN). *N*-acetyl-D-mannosamine is the precursor molecule of sialic acids. Sialic acids are present in the glomerular filtration barrier, functional units of the kidneys, where blood filtration and elimination of metabolic waste occurs. The decrease in these acids is related to membranous nephropathy, minimal lesion disease and the loss of nephrin, a structural protein in podocytes. The synthesis of sialic acid from *N*-acetylneuraminic acid is regulated by the enzyme GNE. The decrease in *N*-acetyl-D-mannosamine precursors may be directly related to the efficiency of the GNE enzyme. Mice with proteinuria and podocytopathy had a good response to treatment when *N*-acetyl-D-mannosamine supplementation was administered (Huizing et al., 2019, Ito et al., 2012).

All the above discussed metabolic pathways for OHN vs. NOHN group comparison (tyrosine, phenylalanine, alanine, and amino sugar and nucleotide sugar metabolism), with exception of the alanine, asparate and glutamate pathways, were also seen in the impact pathway graph for OHN vs. CTR group comparison. The appearance of the same metabolic routes is particularly relevant as it consolidates the hypothesis that these metabolic alterations are indicative of disease progression.

Other metabolites such as alpha ketoglutaric acid, furoylglycine, 4-hydroxyphenylacetic acid, and ascorbic acid were also found altered in the comparisons between OHN vs. NOHN groups and OHN vs. CTR (Table 2). Furthermore, changes in their concentration (increased or decreased) coincide in both comparisons, again in consonance with the disease progression. Reports of unilateral uretheral obstruction in rats as well as patients with acute renal injury indicated an expressive decreased level in urine of ketoglutaric acid and citric acid, intermediary metabolites of Krebs cycle. This decrease has been associated to a possible compromised mitochondrial oxidative metabolism, suggesting lesion of the proximal tubule. Other studies also confirm this observation (Maclellan et al., 2011).

## Conclusion

Although important advances in the evaluation of urinary biomarkers for UPJO have been reported in the literature, none of the discriminant metabolites found so far have managed to be part of tests requested in clinical practice. It is worth mentioning that this work was exploratory in character and it was not designed to find the disease biomarkers. It merely enumerated discriminatory metabolites derived from group comparisons at the disease different stages. Even considering the limitations in size and age variability of this pilot study, 94 urinary metabolites were prospected here. This constitutes an initial effort to reveal promising obstructive HN biomarkers that might in the future contribute to optimize clinical decision-making. When further studies were conducted, with larger cohorts for external validation, including targeted analysis and even biological testing, a biomarker may arise among the discriminatory metabolites found here and hopefully confirm the findings so far established by this work.

## Supplementary Information

Below is the link to the electronic supplementary material.

Supplementary Material 1

## Data Availability

All data supporting the findings of this study are available within the paper and its Supplementary Information.
