# Supplementary material for: Urinary metabolomics profile in children with ureteropelvic junction obstruction
Source: Metabolomics. 2026 Sep 22;22(5):154. doi: 10.1007/s11306-026-02527-0 (PMC13597631; doi:10.1007/s11306-026-02527-0)
Supplement: Supplementary file 1 [file 11306_2026_2527_MOESM1_ESM.docx]

# Urinary metabolomics profile in children with ureteropelvic junction obstruction

**Lucas Henrique Ferreira da Silva, BS. MSc.**

*Center for Multiplatform Metabolomics Studies (CEMM) at the Institute of Chemistry, University of Sao Paulo, Sao Paulo, Brazil.*

**Marcos Figueiredo Mello,^#^ MD. PhD.**

*Division of Urology, Hospital das Clínicas, University of São Paulo Medical School, Sao Paulo, Brazil.*

**Felipe Guilherme Hamoy Kataoka, MD.**

*Department of Surgery, Urology Unit, School of Medicine at the Federal University of Paulo, Sao Paulo, Brazil.*

**Umberto Fauze Amsei Filho, MD.**

*Department of Surgery, Urology Unit, School of Medicine at the Federal University of Sao Paulo, Sao Paulo, Brazil.*

**João Pedro Simon Farah, BS. MSc. PhD.**

*Center for Multiplatform Metabolomics Studies (CEMM) at the Institute of Chemistry, University of Sao Paulo, Sao Paulo, Brazil.*

**Lydia Fumiko Yamaguchi,^##^ BS. MSc. PhD.**

*Institute of Chemistry, University of Sao Paulo, Sao Paulo, Brazil.*

**Massuo Jorge Kato, BS. MSc. PhD.**

*Institute of Chemistry, University of Sao Paulo, Sao Paulo, Brazil.*

**Marcone Augusto Leal de Oliveira, BS. MSc. PhD.**

*Federal University of Juiz de Fora, Juiz de Fora, Brazil.*

**Andréa Tedesco Faccio,^###^ BS. MSc. PhD.**

*Center for Multiplatform Metabolomics Studies (CEMM) at the Institute of Chemistry, University of Sao Paulo, Sao Paulo, Brazil.*

**Lucia Andrade, MD. PhD.**

*Division of Nephrology, Hospital das Clínicas, University of São Paulo Medical School, Sao Paulo, Brazil.*

**Francisco Tibor Dénes, MD. MSc. PhD.**

*Division of Urology, Hospital das Clínicas, University of São Paulo Medical School, Sao Paulo, Brazil.*

**Roberto Iglesias Lopes, MD. PhD.***

*Division of Urology, Hospital das Clínicas, University of São Paulo Medical School, Sao Paulo, Brazil.*

**Marina Franco Maggi Tavares, BS. MSc. PhD.***

*Center for Multiplatform Metabolomics Studies (CEMM) at the Institute of Chemistry, University of Sao Paulo, Sao Paulo, Brazil.*

****Corresponding authors:***

**Marina Franco Maggi Tavares**

Email: mfmtavar@iq.usp.br

Phone: +55 11 3091-2056

**Roberto Iglesias Lopes**

Email: roberto.iglesias@hc.fm.usp.br

Phone: +55 11 3289-1276 ext. 011

***Present Addresses:***

*^#^Department of Surgery, Urology Unit, School of Medicine at the Federal University of Sao Paulo, Sao Paulo, Brazil.*

*^##^Instituto de Pesquisas Tecnológicas do Estado de São Paulo, IPT, NUTABES, Sao Paulo, Brazil.*

^###^*Grupo Fleury, Sao Paulo, Brazil.*

**Table S1.** Metabolites found in the comparison *NOHN vs CTR* by univariate and multivariate statistical analyses.

| **COMPOUND NAME** | **MOLECULAR MASS (Da)** | **MOLECULAR MASS ERROR (ppm)** | **FOLD CHANGE (%)** | **ANALYTICAL TECHIQUE** | **CONFIRMATION** | **STATISTICAL APPROACH** | **adj *p-VALUE (q-VALUE* ) /**  **AUC ( ROC UNIVARIATE)** | **VIP** | **BIOLOGICAL ROUTE** |
| --- | --- | --- | --- | --- | --- | --- | --- | --- | --- |
| 1-Methylhistamine / 3-Methylhistamine * | 126.1025 | 1 | ↑ 97 | HPLC-MS | Putative | Multivariate | **-** | 1.51 | Histidine metabolism |
| 1-Methylhistidine / 3-Methylhistidine * | 192.0743 | 1 | ↑ 101 | HPLC-MS | Putative | Multivariate Univariate | 0.03 (0.072) / 0.749 | 1.52 | Histidine metabolism |
| 1-Methylinosine | 305.088 | 8 | ↓ 52 | HPLC-MS | Putative | Univariate | 0.02 (0.08) / 0.536 |  | Inosine and derivatives (purine metabolism) |
| 2.3-Methylenesuccinic acid | 165.0158 | 0 | ↓ 71 | HPLC-MS | Putative | Univariate | 0.02 (0.06) / 0.768 |  | C5-Branched dibasic acid metabolism |
| 2-Methylcitric acid | 384.1505 | 3 | ↑ 31 | HPLC-MS | Putative | Univariate | 0.01 (0.05) / 0.796 |  | Propanoate metabolism |
| 3-Hydroxy-3-methylglutaric acid | - | - | ↓ 35 | GC-MS (90) | Identified | Univariate | 0.05 (0.09) / 0.660 |  | Lipid metabolism Fatty acid metabolism |
| 3-Hydroxyhippuric acid | 234.0158 | 2 | ↑ 78 | HPLC-MS | Putative | Multivariate | **-** | 1.12 | Fatty acid metabolism |
| 3-Methoxytyrosine | 212.0922 | 1 | ↑ 30 | HPLC-MS | Putative | Multivariate | **-** | 1.50 | Tyrosine and derivatives (Tyrosine metabolism) |
| 4-Hydroxyhippuric acid | 196.0623 | 10 | ↑ 78 | HPLC-MS | Putative | Multivariate | **-** | 1.34 | Fatty acid metabolism |
| Acetylcysteine | 327.0704 | 8 | ↓ 56 | HPLC-MS | Putative | Univariate | 0.01 (0.06) / 0.743 |  | Acid L-cysteine and derivatives (glutathione metabolism) |
| Alpha glucosamine 1-phosphate | - | - | ↓ 49 | GC-MS (80) | Identified | Univariate | 0.01 (0.09) / 0.725 |  | Amino sugar and nucleotide sugar metabolism |
| Aspartylglycosamine | 336.14 | 0 | ↑ 33 | HPLC-MS | Putative | Univariate | 0.01 (0.06) / 0.786 |  | NA |
| Dihyroxy-1H-indole glucuronide | 326.0894 | 6 | ↑ 109 | HPLC-MS | Putative | Multivariate | **-** | 1.06 | NA |
| Dithiothreitol | - | - | ↑ 54 | GC-MS (92) | Identified | Univariate | 0.04 (0.10) / 0.709 | - | Vitamin K metabolism |
| Glucose / Galactose /Fructose (Hexose) * | - | - | ↑ 49 | GC-MS (95) | Identified | Univariate | 0.03 (0.09) / 0.703 | - | NA |
| Glutaminylphenylalanine | 332.0979 | 8 | ↓ 43 | HPLC-MS | Putative | Univariate | 0.03 (0.07) / 0.749 | - | NA |
| Glutamyl-Hydroxyproline (Dipeptideo) | 261.1091 | 2 | ↑ 47 | HPLC-MS | Putative | Multivariate Univariate | 0.01 (0.05) / 0.799 | 1.61 | NA |
| Glutarylcarnitine | 276.1452 | 4 | ↑ 54 | HPLC-MS | Putative | Univariate | 0.03 (0.06) / 0.740 | - | Lipid metabolism pathway |
| Glycylproline (Dipeptide) | 173.0919 | 1 | ↑ 46 | HPLC-MS | Putative | Univariate | 0.02 (0.05) / 0.755 | - | Collagen metabolism |
| Histidine asparagine (Dipeptide) | 292.1035 | 6 | ↑ 36 | HPLC-MS | Putative | Multivariate | **-** | 1.82 | NA |
| Hydroxykynurenine | 263.0417 | 4 | ↑ 43 | HPLC-MS | Putative | Univariate | 0.02 (0.07) / 0.783 | - | Tryptophan metabolism |
| Hydroxyprolyl-Glutamate (Dipeptide) | 261.1091 | 4 | ↑ 47 | HPLC-MS | Putative | Multivariate  Univariate | 0.01 (0.05) / 0.799 | 1.61 | NA |
| Lactobionic acid | - | - | ↑ 52 | GC-MS (86) | Identified | Univariate | 0.04 (0.10) / 0.693 | - | NA |
| Muconic acid | 165.0158 | 0 | ↓ 71 | HPLC-MS | Putative | Univariate | 0.02 (0.06) / 0.768 | - | Benzoate degradation |
| N6.N6-Dimethyl-L-Lysine | 197.1252 | 4 | ↑ 53 | HPLC-MS | Putative | Multivariate | **-** | 1.56 | Lysine degradation |
| N-Acetylneuraminic acid | 332.0979 | 8 | ↓ 43 | HPLC-MS | Putative | Univariate | 0.03 (0.05) / 0.749 | - | Amino Sugar metabolism |
| Pantothenic acid  (Vitamina B5) | - | - | ↓ 49 | GC-MS (78) | Identified | Univariate | 0.03 (0.09) / 0.722 | - | Beta-Alanine metabolism Pantothenate and CoA biosynthesis |
| Pimelic acid | - | - | ↓ 36 | GC-MS (93) | Identified | Univariate | 0.01 (0.06) / 0.732 | - | Fatty acid metabolism Lipid Metabolism |
| Poly-N-acetyllactosamine ( N-acetylglucosamine and galactose) | 384.1505 | 1 | ↑ 31 | HPLC-MS | Putative | Univariate | 0.01 (0.05) / 0.796 | - | NA |
| Prolylglycine (Dipeptide) | 173.0919 | 1 | ↑ 46 | HPLC-MS | Putative | Univariate | 0.02 (0.05) / 0.755 | - | Amino acid degradation pathways |
| Putrescine | 89.1079 | 1 | ↑ 99 | HPLC-MS | Putative | Multivariate | **-** | 1.62 | Arginine and proline metabolism. D-Amino acid metabolism and Glutathione metabolism |
| Salicyluric acid | 196.0623 | 10 | ↑ 78 | HPLC-MS | Putative | Multivariate | **-** | 1.34 | Pathways of salicylate elimination |
| Sorbitol / Mannitol | - | - | ↑ 60 | GC-MS (81/94) | Identified | Univariate | 0.04 (0.09) / 0.702 | - | Fructose and mannose metabolism Galactose metabolism |
| Tagatose | - | - | ↓ 43 | GC-MS (80) | Identified | Univariate | 0.02 (0.09) / 0.712 | - | Galactose metabolism |
| Threonine and Tyrosine (Dipeptide) | 321.0825 | 7 | ↓ 76 | HPLC-MS | Putative | Univariate | 0.02 (0.08) / 0.712 | - | NA |

* analytical method does not distinguish between isomers

NA: non-applicable

**Table S2.** Metabolites found in the comparison *OHN vs NOHN* by univariate and multivariate statistical analyses.

| **COMPOUND NAME** | **MOLECULAR MASS (Da)** | **MOLECULAR MASS ERROR ppm)** | **FOLD CHANGE (%)** | **ANALYTICAL TECHIQUE** | **CONFIRMATION** | **STATISTICAL APPROACH** | **adj *p-VALUE (q-VALUE* ) /**  **AUC ( ROC UNIVARIATE)** | **VIP** | **BIOLOGICAL ROUTE** |
| --- | --- | --- | --- | --- | --- | --- | --- | --- | --- |
| 1.5-anhydri-D-Sorbitol | - | - | ↑ 31 | GC-MS (89) | Identified | Multivariate | **-** | 1.38 | NA |
| 1-Methylhistamine / 3-Methylhistamine * | 126.1025 | 6 | ↓ 47 | HPLC-MS | Putative | Multivariate | **-** | 1.01 | Histidine metabolism |
| 2-Aminomuconic acid | 158.0439 | 6 | ↓ 35 | HPLC-MS | Putative | Univariate | 0.01 (0.10) / 0.775 | 1.0 | Tryptophan metabolism |
| 2-Methylbutyrylglycine | 198.0524 | 2 | ↑ 93 | HPLC-MS | Putative | Univariate | 0.02 (0.05) / 0.794 | - | Pathway of L- isoleucine |
| 3-(3-Hydroxyphenyl)propanoic acid | 205.0249 | 6 | ↑ 147 | HPLC-MS | Putative | Multivariate | **-** | 1.79 | Phenylalanine metabolism |
| 4-Hydroxy-3-methylbenzoic acid | 153.054 | 9 | ↑ 124 | HPLC-MS | Putative | Multivariate | **-** | 1.32 | NA |
| 4-hydroxybenzoic acid | - | - | ↑ 96 | GC-MS (85) | Identified | Multivariate Univariate | 0.04 (0.09) / 0.727 | 1.22 | Ubiquinone and other terpenoid-quinone biosynthesis |
| 4-hydroxyphenylacetic acid | 153.054 | 6 | ↑ 124 | HPLC-MS | Putative | Multivariate | **-** | 1.32 | Tyrosine metabolism and Phenylalanine metabolism |
|  |  | - | ↑ 112 | GC-MS (96) | Identified | Multivariate Univariate | 0.0006 (0.05) / 0.90 | 1.97 |  |
| 4-Methoxyphenylacetic acid | 205.0249 | 6 | ↑ 147 | HPLC-MS | Putative | Multivariate | **-** | 1.79 | Tyrosine metabolism and Phenylalanine metabolism |
| 5-Aminopentanoic acid | 235.1659 | 3 | ↓ 46 | HPLC-MS | Putative | Univariate | 0.02 (0.06) / 0.712 |  | Lysine degradation |
| 5-Hydroxyindoleacetaldehyde | 176.0707 | 1 | ↑ 96 | HPLC-MS | Putative | Multivariate | 0.02 (0.05) / 0.770 | 1.62 | Tryptophan metabolism |
| 6-Methylsalicylic acid | 153.054 | 9 | ↑ 124 | HPLC-MS | Putative | Multivariate | **-** | 1.32 | Biosynthesis of enediyne antibiotics |
| Adenine | 158.0439 | 1 | ↓ 35 | HPLC-MS | Putative | Univariate | 0.01 (0.11) / 0.775 | - | Purine metabolism |
| Alpha ketoglutaric acid | - | - | ↓ 47 | GC-MS (79) | Identified | Multivariate | **-** | 1.26 | Citrate cycle (TCA cycle) |
| Alpha-glucosamine 1-phosphate | - | - | ↑ 48 | GC-MS (80) | Identified | Multivariate | **-** | 1.46 | Amino sugar and nucleotide sugar metabolism |
| Asparagine alanine / Glutamine glycine (Dipeptide) | 226.0808 | 4 | ↓ 35 | HPLC-MS | Putative | Multivariate | **-** | 1.27 | Citrate cycle (TCA cycle) |
| Beta- alanine | - | - | ↓ 36 | GC-MS (96) | Identified | Multivariate | **-** | 1.01 | Beta-Alanine metabolism |
| Cellobiose (Disaccharides) | - | - | ↓ 46 | GC-MS (90) | Identified | Multivariate | **-** | 1.36 | NA |
| Desaminotyrosine | 205.0249 | 6 | ↑ 147 | HPLC-MS | Putative | Multivariate | **-** | 1.79 | Phenylalanine metabolism and Tyrosine metabolism |
| D-Fucose/2-Deoxyglucose (Hexose)* | 187.0589 | 3 | ↑ 102 | HPLC-MS | Putative | Multivariate | **-** | 1.63 | NA |
| D-glucose (hexose) | - | - | ↑ 30 | GC-MS (97) | Identified | Multivariate | **-** | 1.05 | NA |
| D-threitol | - | - | ↓ 30 | GC-MS (97) | Identified | Multivariate Univariate | 0.006 (0.09) / 0.807 | 1.32 | NA |
| Furoylglycine | - | - | ↓ 64 | GC-MS (85) | Identified | Multivariate Univariate | 0.01 (0.09) / 0.807 | - | Fatty acid beta-oxidation |
| Gluconic acid | - | - | ↓ 39 | GC-MS (90) | Identified | Multivariate Univariate | 0.009 (0.05) / 0.813 | - | Pentose phosphate pathway |
| Glucuronic acid | - | - | ↓ 42 | GC-MS (85) | Identified | Multivariate | **-** | 1.16 | Aminobenzoate degradation |
| Glutamine hydroxyproline (Dipeptide) | 298.0789 |  | ↓ 31 | HPLC-MS | Putative | Multivariate | **-** | 1.37 | NA |
| Glycerol 1-phosphate | - | - | ↑ 36 | GC-MS (84) | Identified | Multivariate | **-** | 1.04 | Glycerolipid metabolism |
| Hydroxykynurenine | 263.0417 | 4 | ↓ 36 | HPLC-MS | Putative | Univariate | 0.02 (0.07) / 0.760 | - | Tryptophan metabolism |
| Indole-3-acetic-acid-O-glucuronide | 390.0621 | 9 | ↓ 40 | HPLC-MS | Putative | Univariate | 0.02 (0.07) / 0.612 | - | NA |
| Indoleacetic acid | 176.0707 | 1 | ↑ 96 | HPLC-MS | Putative | Univariate | 0.02 (0.05)/ 0.770 | - | Tryptophan metabolism |
| L-3-Phenyllactic acid | 205.0249 | 6 | ↑ 147 | HPLC-MS | Putative | Multivariate | **-** | 1.79 | Phenylalanine metabolism |
| Lactobionic acid | - | - | ↓ 37 | GC-MS (86) | Identified | Univariate | 0.01 (0.09)/ 0.760 | - | NA |
| L-ascorbic acid | - | - | ↑ 110 | GC-MS (83) | Identified | Multivariate Univariate | 0.04 (0.05) / 0.753 | - | Ascorbate and aldarate metabolism |
| L-Valine | 235.1659 | 3 | ↓ 46 | HPLC-MS | Putative | Univariate | 0.02 (0.06) / 0.712 | - | Valine. leucine and isoleucine degradation |
| Mandelic Acid | 153.054 | 9 | ↑ 124 | HPLC-MS | Putative | Multivariate | **-** | 1.32 | Aminobenzoate degradation |
| Methyl (E)-2-dodecenoate | 235.1659 | 4 | ↓ 46 | HPLC-MS | Putative | Univariate | 0.02 (0.06) / 0.712 | - | Lipid metabolism |
| N-acetyl-D-mannosamine | - | - | ↓ 38 | GC-MS (89) | Identified | Multivariate | **-** | 1.15 | Amino sugar and nucleotide sugar metabolism |
| N-acetyl-L-aspartic acid | 176.0708 | 1 | ↑96 | HPLC-MS | Putative | Univariate | 0.02 (0.05) / 0.770 | - | Alanine. aspartate and glutamate metabolism |
|  |  | - | ↑50 | GC-MS (81) | Identified | Multivariate | - | 1.26 |  |
| Ribose/ Arabinose / Xylose (Pentose)* | - | - | ↑ 114 | GC-MS (91) | Identified | Multivariate | **-** | 1.71 | NA |
| Sorbitol/ Mannitol * | - | - | ↓ 50 | GC-MS (90/94) | Identified | Univariate | 0.02 (0.09)/ 0.780 | - | Fructose and mannose metabolism |

* analytical method does not distinguish between isomers

NA: non-applicable

**Table S3.** Metabolites found in the comparison *OHN vs CTR* by univariate and multivariate statistical analyses.

| **COMPOUND NAME** | **MOLECULAR MASS (Da)** | **MOLECULAR MASS ERROR (ppm)** | **FOLD CHANGE (%)** | **ANALYTICAL TECHIQUE** | **CONFIRMATION** | **STATISTICAL APPROACH** | **adj *p-VALUE***  ***(q-VALUE* ) /**  **AUC ( ROC UNIVARIATE)** | **VIP** | **BIOLOGICAL ROUTE** |
| --- | --- | --- | --- | --- | --- | --- | --- | --- | --- |
| 12-Oxo-2.3-dinor-10.15-phytodienoic acid | 303.1351 | 2 | ↑ 73 | HPLC-MS | Putative | Univariate | 0.003 (0.06) / 0.866 | - | Lipid metabolism pathway |
| 2-(6-carboxy-3.4.5-trihydroxyoxan-2-yl)propanedioic acid | 319.0073 | 3 | ↓ 65 | HPLC-MS | Putative | Multivariate | **-** | 2.53 | NA |
| 2-Methylbutyrylglycine | 198.0524 | 2 | ↑ 141 | HPLC-MS | Putative | Univariate | 0.002 (0.05) / 0.882 | - | NA |
| 2-Methylcitric acid | 341.1505 | 3 | ↑ 75 | HPLC-MS | Putative | Univariate | 0.001 (0.04) / 0.845 | - | Propanoate metabolism |
| 2-Phenylglycine | 303.1351 | 4 | ↑ 73 | HPLC-MS | Putative | Univariate | 0.003 (0.06) / 0.866 | - | NA |
| 3-(2-hydroxyphenyl)propanoic acid | 205.0249 | 6 | ↑ 258 | HPLC-MS | Putative | Multivariate | **-** | 1.63 | Phenylalanine metabolism |
| 3.7-Dimethyl-2.6-octadien-1-ylacetate | 219.1338 | 8 | ↓ 25 | HPLC-MS | Putative | Multivariate | **-** | 1.26 | Lipid metabolism pathway |
| 3-Cresotinic acid | 153.054 | 4 | ↑ 132 | HPLC-MS | Putative | Univariate | 0.02 (0.06) / 0.807 | - | NA |
| 3-Hydroxy-3-methylglutaric acid (dicrotalic acid) | - | - | ↓ 43 | GC-MS (90) | Identified | Multivariate Univariate | 0.04 (0.09) / 0.770 | - | Lipid metabolism Fatty acid metabolism |
| 3-Hydroxy-4-methoxymandelate | 221.0406 | 7 | ↓ 46 | HPLC-MS | Putative | Multivariate | **-** | 2.03 | NA |
| 3-Hydroxyphenylacetate | 153.054 | 4 | ↑ 132 | HPLC-MS | Putative | Univariate Multivariate(*) | 0.02 (0.06) / 0.807 | 1.60 | Tyrosine metabolism |
| 3-Methoxy-4-hydroxymandelate | 221.0406 | 7 | ↓ 46 | HPLC-MS | Putative | Univariate | 0.02 (0.05) / 0.813 | - | Tyrosine metabolism |
| 3-Methoxyphenylacetic acid | 205.0249 | 6 | ↑ 258 | HPLC-MS | Putative | Multivariate | **-** | 1.63 | NA |
| 3-Methoxytyramine | 206.0595 | 8 | ↑ 109 | HPLC-MS | Putative | Multivariate | **-** | 1.39 | Tyrosine metabolism |
| 3-Phenyllactic acid | 205.0249 | 6 | ↑ 258 | HPLC-MS | Putative | Multivariate | **-** | 1.63 | Phenylalanine metabolism |
| 4-acetamidobutyric acid | - | - | ↓ 32 | GC-MS (90) | Identified | Multivariate | **-** | 1.16 | Arginine and proline metabolism |
| 4-hydroxy-2.3-dimethoxybenzoic acid | 221.0406 | 7 | ↓ 46 | HPLC-MS | Putative | Univariate | 0.02 (0.05) / 0.813 | - | NA |
| 4-Hydroxy-3-methylbenzoic acid | 153.054 | 4 | ↑ 137 | HPLC-MS | Putative | Univariate Multivariate(*) | 0.02 (0.06) / 0.807 | 1.60 | NA |
| 3-Hydroxyphenylacetate | 153.054 | 4 | ↑132 | HPLC-MS | Putative | Univariate | 0.02 (0.06) / 0.807 | - | Tyrosine metabolism |
|  |  |  | ↑84 | GC-MS (96) | Identified | Multivariate | **-** | 1.66 |  |
| 5.6-Dihydrouridine | 247.093 | 2 | ↑ 68 | HPLC-MS | Putative | Multivariate | **-** | 1.47 | NA |
| 5-Hydroxyindoleacetaldehyde | 176.0707 | 1 | ↑ 139 | HPLC-MS | Putative | Univariate | 0.004 (0.05) / 0.872 | - | Tryptophan metabolism |
| 5-Hydroxylysine | 163.1063 | 9 | ↑ 137 | HPLC-MS | Putative | Multivariate | **-** | 1.62 | Lysine degradation |
| 6-Methylmercaptopurine | 84.0232 | 3 | ↑ 39 | HPLC-MS | Putative | Multivariate | **-** | 1.02 | Mercaptopurine Metabolism Pathway |
| 6-Methylsalicylic acid | 153.054 | 4 | ↑ 137 | HPLC-MS | Putative | Univariate Multivariate(*) | 0.02 (0.06) / 0.807 | 1.60 | Biosynthesis of enediyne antibiotics |
| Alpha ketoglutaric acid | - | - | ↓ 49 | GC-MS (79) | Identified | Multivariate | **-** | 1.66 | Citrate cycle (TCA cycle) |
| Arabitol / Ribitol / Xylitol (Pentoses alcohol) * | - | - | ↑ 81 | GC-MS (94) | Identified | Multivariate Univariate | 0.001 (0.05) / 0.882 | - | NA |
| Ascorbic acid | - | - | ↑ 55 | GC-MS (83) | Identified | Multivariate | **-** | 1.18 | Ascorbate and aldarate metabolism |
| Asparagine alan  ine / Glutamine glycine (Dipeptide) | 226.0808 | 4 | ↓ 42 | HPLC-MS | Putative | Multivariate | **-** | 1.48 | NA |
| Aspartylhydroxyproline (Dipeptide) | 247.093 | 2 | ↑ 69 | HPLC-MS | Putative | Multivariate | **-** | 1.47 | Breakdown product of collagen |
| Citric acid | 230.9886 | 7 | ↓ 40 | HPLC-MS | Putative | Multivariate | **-** | 1.57 | Citrate cycle (TCA cycle) |
| Cystathionine | 223.0742 | 2 | ↑ 182 | HPLC-MS | Putative | Multivariate | **-** | 2.24 | Glycine. serine and threonine metabolism |
| Cystine | 241.0308 | 1 | ↑ 156 | HPLC-MS | Putative | Multivariate | **-** | 1.63 | Cysteine and methionine metabolism |
| Desaminotyrosine | 205.0249 | 6 | ↑ 258 | HPLC-MS | Putative | Multivariate | - | 1.63 | Tyrosine metabolism |
| Diketogulonic acid | 230.9886 | 7 | ↓ 40 | HPLC-MS | Putative | Multivariate | **-** | 1.57 | Pentose and glucuronate interconversions |
| D-Threitol | 145.0474 | 2 | ↑ 57 | HPLC-MS | Putative | Multivariate | **-** | 1.16 | NA |
| Erythritol - Uremia | 145.0474 | 2 | ↑ 57 | HPLC-MS | Putative | Multivariate | **-** | 1.16 | ABC transporters |
| Fucose (Hexose) * | - | - | ↑ 55 | GC-MS (80) | Identified | Multivariate | **-** | 1.18 | NA |
| Furoylglycine | 192.0272 | 2 | ↓55 | HPLC-MS | Putative | Multivariate | **-** | 1.62 | Fatty acid beta-oxidation |
|  |  |  | ↓51 | GC-MS (89) | Identified | Univariate | 0.03 (0.09) / 0.662 | - |  |
| Galabiose -Maltose -Trehalose (Disaccharides)* | 365.1053 | 0 | ↓ 20 | HPLC-MS | Putative | Multivariate | **-** | 1.63 | Starch and sucrose metabolism |
| Gemcitabine diphosphate | 212.5101 | 3 | ↓ 56 | HPLC-MS | Putative | Multivariate | **-** | 2.18 | NA |
| Geranyl phenylacetate | 273.1842 | 3 | ↑ 80 | HPLC-MS | Putative | Multivariate | **-** | 2.80 | Lipid metabolism pathway |
| Gluconic acid | - | - | ↓ 54 | GC-MS (90) | Identified | Multivariate | **-** | 1.24 | Carbon metabolism |
| Glutaminylglutamic acid (Dipeptide) | 276.1183 | 3 | ↑ 84 | HPLC-MS | Putative | Univariate | 0.01 (0.07) / 0.761 | - | NA |
| Glutamyl-Hydroxyproline | 261.1091 | 4 | ↑ 73 | HPLC-MS | Putative | Multivariate Univariate | 0.001 (0.05) / 0.877 | 1.58 | Amino acid degradation pathways |
| Glycylproline (Dipeptide) | 173.0919 | 1 | ↑ 40 | HPLC-MS | Putative | Univariate | 0.02 (0.05) / 0.834 | - | Collagen metabolism |
| Hydroxyprolyl-Proline (Dipeptide) | 229.1196 | 6 | ↑ 72 | HPLC-MS | Putative | Univariate | 0.02 (0.05) / 0.749 | - | Incomplete breakdown product of protein digestion or protein catabolism |
| Hydroxyvalerylcarnitine | 302.1342 | 7 | ↑ 41 | HPLC-MS | Putative | Multivariate | **-** | 1.14 | Lipid metabolism pathway |
| Indoleacetic acid | 176.0707 | 1 | ↑ 139 | HPLC-MS | Putative | Univariate | 0.004 (0.05) / 0.872 | - | Tryptophan metabolism |
| Leucrose | - | - | ↓ 50 | GC-MS (97) | Identified | Multivariate | **-** | 1.06 | NA |
| Maltose/ Trehalose (Disaccharides) | - | - | ↑ 36 | GC-MS (96) | Identified | Multivariate | **-** | 1.54 | Starch and sucrose metabolism |
| Mandelic Acid | 153.054 | 4 | ↑ 137 | HPLC-MS | Putative | Univariate Multivariate(*) | 0.02 (0.06) / 0.807 | 1.60 | Aminobenzoate degradation |
| Mannitol/Sorbitol/Galactiol/ Iditol (Sugars acid) * | 221.0406 | 7 | ↓ 46 | HPLC-MS | Putative | Multivariate Univariate | 0.02 (0.05) / 0.813 | - | Fructose and mannose metabolism |
| Methionine sulfoxide | 166.0534 | 9 | ↑ 152 | HPLC-MS | Putative | Multivariate | **-** | 1.62 | Cysteine and methionine metabolism |
| Myo-Inositol | 219.0273 | 3 | ↓ 31 | HPLC-MS | Putative | Multivariate | **-** | 1.08 | Ascorbate and aldarate metabolism |
| N-gamma-Glutamylglutamine (Dipeptide) | 276.1183 | 3 | ↑ 84 | HPLC-MS | Putative | Univariate | 0.01 (0.07) / 0.761 | - | Glutamine and derivatives |
| Pantothenic acid (Vitamina B5) | - | - | ↓ 59 | GC-MS (77) | Identified | Multivariate Univariate | 0.005 (0.09) / 0.833 | - | Beta-Alanine metabolism Pantothenate and CoA biosynthesis |
| Poly-N-acetyllactosamine ( N-acetylglucosamine and galactose) | 384.1505 | 1 | ↑ 75 | HPLC-MS | Putative | Univariate | 0.01 (0.05) / 0.796 | - | NA |
| Prolylglycine (Dipeptide) | 173.0919 | 1 | ↑ 39 | HPLC-MS | Putative | Univariate | 0.02 (0.05) / 0.834 | - | Incomplete breakdown product of protein digestion or protein catabolism. |
| Putrescine | 89.1079 | 6 | ↑ 283 | HPLC-MS | Putative | Multivariate | **-** | 2.36 | Arginine and proline metabolism |
| Ribose (Pentose) | - | - | ↑ 30 | GC-MS (91) | Identified | Multivariate | **-** | 1.26 | NA |
| Sorbose/Allose/Fructose/ Glucose/Tagatose(Hexose)* | - | - | ↓ 64 | GC-MS (92) | Identified | Multivariate | **-** | 1.07 | NA |
|  | 219.0273 | 3 | ↓ 31 | HPLC-MS | Putative | Multivariate | **-** | 1.08 | NA |
| Syringic acid | 221.0406 | 7 | ↓ 46 | HPLC-MS | Putative | Univariate | 0.02 (0.05) / 0.813 | - | Aminobenzoate degradation |
| Tagatose (Pentose) | - | - | ↓ 48 | GC-MS (80) | Identified | Multivariate Univariate | 0.04 (0.09) / 0.721 | 1.10 | NA |
| Taurine | 126.0211 | 7 | ↑ 99 | HPLC-MS | Putative | Multivariate | **-** | 1.15 | Taurine and hypotaurine metabolism |
| Threonic acid | - | - | ↓ 32 | GC-MS (97) | Identified | Multivariate | **-** | 1.03 | Ascorbate and aldarate metabolism |
| Uric acid | 191.018 | 2 | ↑ 78 | HPLC-MS | Putative | Multivariate | **-** | 1.32 | Purine metabolism |

* analytical method does not distinguish between isomers

NA: non-applicable
